# Assessment of thoron contribution to indoor radon exposure in Canada

**DOI:** 10.1007/s00411-021-00956-0

**Published:** 2022-01-01

**Authors:** Jing Chen

**Affiliations:** Radiation Protection Bureau, Health Canada, 775 Brookfield Road, Ottawa, K1A1C1 Canada

**Keywords:** Rn-220, Indoor, Radon

## Abstract

From 2007 to 2013, simultaneous radon (^222^Rn) and thoron (^220^Rn) measurements were conducted in a total of 3534 residential homes in 34 metropolitan areas covering 71% of the Canadian population. While radon levels were above the detector’s detection limit in almost all homes, thoron concentrations were measurable in only 1738 homes. When analysis was limited to homes where thoron concentrations exceeded the detection limit, a pooled analysis confirmed that thoron is log-normally distributed in the indoor environment, and the distribution was characterized by a population-weighted geometric mean of 13 Bq/m^3^ and a geometric standard deviation of 1.89. Thoron contribution to indoor radon dose varied widely, ranging from 1.3 to 32% geographically. This study indicated that on average, thoron contributes 4% of the radiation dose due to total indoor radon exposure (^222^Rn and ^220^Rn) in Canada.

## Introduction

Radon is a naturally occurring radioactive gas generated by the decay of uranium- and thorium-bearing minerals in rocks and soils. Radon and its decay products are the major contributors to human exposure from natural radiation sources (UNSCEAR 2000, 2010). Radon has been identified as the second leading cause of lung cancer after tobacco smoking (WHO 2009; UNSCEAR 2019). The most common isotopes of radon are ^222^Rn (radon gas) and ^220^Rn (thoron gas). ^222^Rn is a product of the natural radioactive decay of uranium whereas ^220^Rn is a product of the decay of thorium. Both uranium and thorium are found as trace elements in most rocks and soil in varying concentrations across Canada, as shown on the radioactivity map of Canada (Geological Survey of Canada 2014).

Following the revision of the Canadian radon guideline from 800 to 200 Bq/m^3^ in June 2007, a number of regional and national radon and thoron surveys were conducted. This study summarizes data from simultaneous radon and thoron surveys conducted in a number of communities between 2007 and 2013 to provide information on thoron distribution characteristics across Canada, and to assess the contribution of thoron exposure (^220^Rn) to the total indoor radon exposure (^220^Rn and ^222^Rn). Furthermore, this assessment revisits the treatment of thoron measurements below the detection limit that was used to assess average thoron exposure in a previous study (Chen et al. 2015).

## Materials and methods

In 2012, Health Canada launched a survey to simultaneously measure radon (^222^Rn) and thoron (^220^Rn) in residential homes in 33 metropolitan areas across Canada (Chen et al. 2015). This survey was preceded by smaller studies conducted in Ottawa, Winnipeg, Fredericton and Halifax between 2007 and 2010 (Chen et al. 2008, 2009, 2011a). Original data from all these measurement campaigns were pooled in the assessment described in the present paper to provide coverage for 34 metropolitan areas (Fredericton was not included in the 2012 survey), and analyzed as described below.

All survey data summarized here are from long-term measurements collected using passive integrated radon–thoron discriminative detectors developed at the National Institute of Radiological Sciences (NIRS) in Japan (commercially known as RADUET). A RADUET contains paired detection chambers: a low-diffusion chamber and a high-diffusion chamber. The low-diffusion chamber limits diffusion of thoron into the chamber while the high-diffusion chamber is designed such that both radon and thoron can diffuse into the chamber easily. The lower detection limits (DLs) of RADUETs were determined to be 3 Bq/m^3^ for radon and 4 Bq/m^3^ for thoron (Chen et al. 2008).

Using two alpha track densities (TD) of low and high-diffusion chambers (TD_L_, TD_H_), radon and thoron concentrations (*C*_Rn_, *C*_Tn_) can be obtained by solving the following equations (Zhuo et al. 2002; Tokonami et al. 2005):

1 $${\mathrm{TD}}_{\mathrm{L}}={c}_{11}\left({C}_{\mathrm{Rn}}-{b}_{1}\right)+{c}_{12}\left({C}_{\mathrm{Tn}}-{b}_{2}\right),$$

2 $${\mathrm{TD}}_{\mathrm{H}}={c}_{21}\left({C}_{Rn}-{b}_{1}\right)+{c}_{22}\left({C}_{Tn}-{b}_{2}\right),$$

where *b*_1_ and *b*_2_ are the background noise levels for radon and thoron concentration, respectively, i.e., they appear as radon or thoron concentration readings for blank control detectors. The values *c*_11_*, c*_12_*, c*_21_ and *c*_22_ are the calibration coefficients.

RADUET detectors purchased at different times were calibrated before deploying and analyzing the detectors. The calibration was done by randomly choosing groups of RADUETs and exposing them to three different known radon and thoron concentrations at NIRS and Hirosaki University in Japan. This calibration determined the coefficients in Eqs. (1) and (2), to ensure the quality of radon and thoron measurements by accounting for possible changes in the detectors after the storage period and variations in the reader system and/or etching process.

The annual effective doses due to indoor radon and thoron exposure for each metropolitan area were assessed based on the formula given by the United Nations Scientific Committee on the Effects of Atomic Radiation (UNSCEAR) (UNSCEAR 2000, 2010):

3 $${E}_{\mathrm{Rn}}={C}_{\mathrm{Rn}}\times {F}_{\mathrm{Rn}}\times 7000\times 9,$$

4 $${E}_{\mathrm{Tn}}={C}_{\mathrm{Tn}}\times {F}_{\mathrm{Tn}}\times 7000\times 40,$$

where *C*_Rn_ and *C*_Tn_ are arithmetic mean (AM) concentrations (in Bq/m^3^) for indoor radon and thoron gas, respectively, and *F*_Rn_ and *F*_Tn_ are equilibrium factors for indoor radon and thoron, respectively. For annual dose calculation, an 80% home occupancy time, i.e., 7000 h, was assumed. The dose conversion factors are 9 and 40 nSv/(h Bq/m^3^) for radon and thoron, respectively (UNSCEAR 2000).

Radon and thoron as well as their progenies are present in the indoor atmosphere as attached and unattached fractions. The level of ventilation in a house and the plating out of radon/thoron progenies onto surfaces determine the extent of equilibrium (or disequilibrium) between radon/thoron gases and their progenies. As gases, radon and thoron contribute very little to the dose to the lung. It is the inhalation of the short-lived, solid decay products and their subsequent deposition on the walls of the airway epithelium of the bronchial tree that delivers most of the radiation dose to human lungs (Harley 2018). Presently, direct measurements of the concentrations of all short-lived decay products of ^222^Rn and ^220^Rn are limited. The progeny concentrations, *C*_RnP_ and *C*_TnP_, are often estimated from considerations of equilibrium between these isotopes and their respective decay products by multiplying the gas concentration with the local specific equilibrium factor; in other words, *C*_RnP_ = *F*_Rn_*·C*_Rn_ and *C*_TnP_ = *F*_Tn_*·C*_Tn_. In the present assessment, Canadian-specific equilibrium factors of *F*_Rn_ = 0.54 and *F*_Tn_ = 0.022 are used (Chen and Marro 2011; Chen et al. 2011b, 2012a).

Thoron contribution to radon exposure in Canadian homes is determined by dividing the annual effective dose of thoron exposure (*E*_Tn_) by the annual effective dose of the total radon exposure (*E*_Tn_ and *E*_Rn_):

5 $${R}_{\mathrm{Tn}}=\frac{{E}_{\mathrm{Tn}}}{{E}_{\mathrm{Rn}}+{E}_{\mathrm{Tn}}}$$

Previous studies in Ottawa, Winnipeg, Halifax and Fredericton indicated that thoron concentration is log-normally distributed in indoor environment (Chen et al. 2008, 2009, 2011a). Therefore, the geometric mean (GM) and geometric standard deviation (GSD) are calculated to characterize thoron concentration distribution in residential homes.

## Results and discussion

Results from a total of 3534 measurements in residential homes in 34 metropolitan areas are summarized in Table 1. While radon levels were above the DL (3 Bq/m^3^) in almost all homes, thoron concentrations were below the DL (4 Bq/m^3^) in 50% of homes tested. In all surveyed areas, both radon and thoron concentrations varied widely. The highest radon concentration of 2341 Bq/m^3^ was measured in Halifax area, and the highest thoron concentration of 1977 Bq/m^3^ was found in the city of Fredericton.

**Table 1:** Number of reported results, numbers (counts) of radon and thoron concentrations below detection limits, minimum and maximum measured radon and thoron concentrations in 34 metropolitan areas

Metropolitan area	Population*	Results reported	Counts *C*_Rn_ < DL	Counts *C*_Tn_ < DL	*C*_Rn_ min	*C*_Rn_ max	*C*_Tn_ min	*C*_Tn_ max
Abbotsford-Mission	180,518	90	1	38	< 3	466	< 4	136
Barrie	197,059	88	0	44	11	763	< 4	134
Brantford	134,203	89	2	43	< 3	472	< 4	110
Calgary	1,392,609	99	0	64	18	850	< 4	26
Edmonton	1,321,426	97	0	51	13	386	< 4	45
Fredericton	58,220	45	0	28	16	1374	< 4	1977
Greater Sudbury	164,689	96	0	56	18	748	< 4	103
Guelph	151,984	102	0	59	18	982	< 4	89
Halifax–Dartmouth	495,691	165	0	86	4	2341	< 4	206
Hamilton	747,545	87	0	42	18	397	< 4	180
Kelowna	194,882	108	1	63	< 3	1146	< 4	72
Kingston	161,175	97	0	53	12	703	< 4	146
Kitchener–Cambridge–Waterloo	523,894	102	0	55	21	211	< 4	172
London	494,069	81	0	43	15	340	< 4	43
Moncton	144,810	95	1	47	< 3	660	< 4	75
Montréal	4,098,927	99	1	48	< 3	772	< 4	48
Oshawa	379,848	96	1	42	< 3	322	< 4	110
Ottawa—Gatineau	1,323,783	204	0	82	8	1525	< 4	924
Peterborough	121,721	100	0	52	22	334	< 4	30
Québec	800,296	99	0	58	6	956	< 4	199
Regina	236,481	96	0	69	29	1335	< 4	78
Saguenay	160,980	100	1	44	< 3	700	< 4	197
Saint John	126,202	116	0	54	6	815	< 4	205
Saskatoon	295,095	104	0	65	43	460	< 4	86
Sherbrooke	212,105	104	0	60	19	1672	< 4	47
St. Catharines—Niagara	406,074	87	1	32	< 3	172	< 4	63
St. John’s	205,955	97	1	52	< 3	330	< 4	27
Thunder Bay	121,621	94	2	53	< 3	607	< 4	121
Toronto	5,928,040	91	0	33	7	182	< 4	56
Trois-Rivières	156,042	95	1	30	< 3	101	< 4	98
Vancouver	2,463,431	98	0	40	6	210	< 4	34
Victoria	367,770	102	0	46	4	174	< 4	44
Windsor	329,144	94	0	55	18	742	< 4	210
Winnipeg	778,489	217	0	109	20	1220	< 4	297
Total	24,874,778	3534	13	1796	< 3	2341	< 4	1977

*DL* detection limit, *C*_*Rn*_ concentration (in Bq/m^3^) for indoor radon gas, *C*_*Tn*_ concentration (in Bq/m^3^) for indoor thoron gas

*Populations are taken from Census 2016 (Statistics Canada 2020)

In studies to characterize the thoron equilibrium factor in Canadian homes (Chen et al. 2011b, 2012a), thoron progeny detectors, developed at NIRS in Japan, were deployed side-by side with RADUET radon/thoron gas detectors. These surveys were conducted in a total of 247 homes in the cities of Ottawa, Winnipeg, Halifax and Fredericton. While thoron progeny concentrations, *C*_TnP_, were recorded in all progeny detectors, only 113 homes had thoron gas concentrations above the DL. It is of interest to examine whether there are significant changes in the characteristics if the given *C*_TnP_ values were grouped into two: one group that contains the cases where thoron gas concentrations were below DL and another group that consists of the cases where thoron gas concentrations could be measured. The characteristics of *C*_TnP_ for those two groups are listed in Table 2. On average, thoron progeny concentrations in houses of group 1 were somewhat lower than those of group 2. However, considering the wide variation ranges and limited sample sizes, thoron progeny distribution characteristics are generally similar for the two groups; GM = 0.44 Bq/m^3^ with GSD = 2.52 for group 1, and GM = 0.59 Bq/m^3^ with GSD = 2.56 for group 2.

**Table 2:** Characteristics of thoron progeny concentration *C*_TnP_ in units of Bq/m^3^ for group 1 with undetectable *C*_Tn_ and group 2 with detectable *C*_Tn_ in units of Bq/m^3^

	Samples	AM	*C*_Tn_			*C*_TnP_	
			GM	GSD	AM (range)	GM	GSD
Group 1, *C*_Tn_ < DL	134	–	–	–	0.73 (0.071, 7.45)	0.44	2.52
Group 2, *C*_Tn_ ≥ DL	113	60	27	2.94	0.99 (0.077, 8.03)	0.59	2.56

*AM* arithmetic mean, *GM* geometric mean, *GSD* geometric standard deviation

Thoron gas has a relatively short physical half-life of 55.6 s. This means that thoron does not have much time to travel from its production site to the immediate environment of human beings, or to a detector. However, the relatively long physical half-life of one of its decay products, ^212^Pb (10.6 h), allows this isotope enough time to migrate away from its source before decaying further and producing the important alpha-emitter ^212^Bi (half-life 60.6 min) and ^212^Po (half-life 0.3 μs). This suggests that thoron progenies could be rather homogeneously distributed in the indoor air, while thoron gas concentration will likely be more localized relative to its sources. In this case, the thoron gas concentrations measured by a detector such as a RADUET could be significantly affected by ability of air from the source to reach the device in less than a minute, and the resulting thoron measurement may not be representative of the concentration of alpha energy in indoor air, attributable to thoron progenies.

Based on the results shown in Table 2, even when thoron gas was undetected, the average progeny concentration was only slightly lower than the average progeny concentration when thoron gas was measurable. However, the difference could be well within the uncertainty range for the limited sample sizes of the two groups. Even so, this evidence does not support assigning thoron concentration at half of the DL to results below DL.

Thoron equilibrium factor, *F*_Tn_, was determined using only measurements where both thoron gas and progeny concentrations were known. Therefore, when using *F*_Tn_ to convert thoron gas concentration to thoron progeny concentration for the calculation of effective dose (Eq. 2), thoron gas concentrations below DL should not be included in the statistical analysis. In the subsequent analysis, only those paired results of radon and thoron with *C*_Tn_ > DL are considered.

**Fig. 1:**
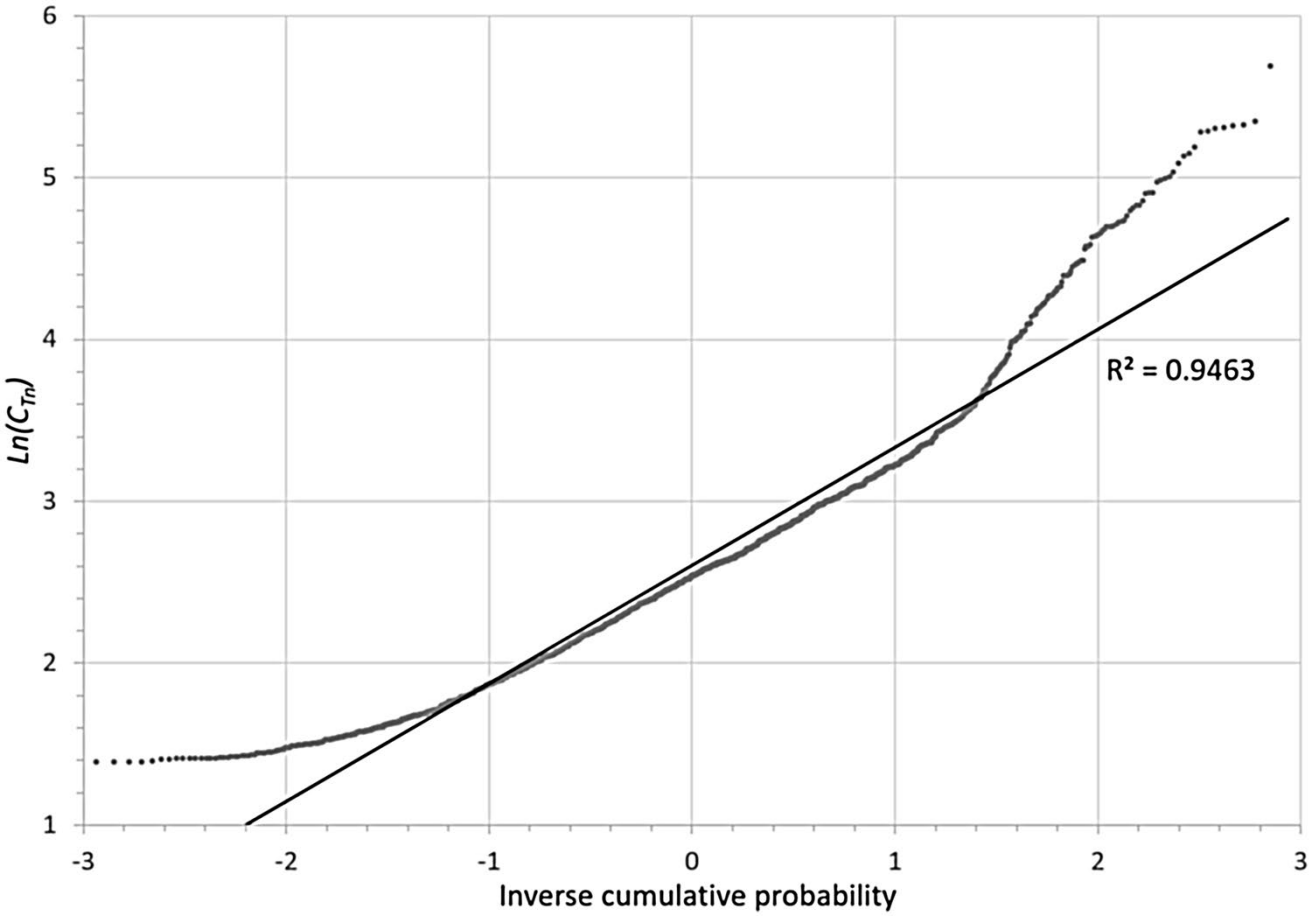
Inverse cumulative probability distribution of thoron concentrations (*C*_Tn_) in 1738 homes of the 34 metropolitan areas listed in Table 1

With a total of 1738 thoron results above DL, a cumulative probability test indicated that the data are log-normally distributed, as shown in Fig. 1. The *x*-axis shows the inverse cumulative probability and the *y*-axis gives the natural logarithms of thoron gas concentration *C*_Tn_ in Bq/m^3^. The majority of data points falling near a straight line implies that a log-normal distribution will fit the data with good fidelity and that the geometric mean (GM) and geometric standard deviation (GSD) are the appropriate parameters to characterize the average annual residential thoron concentrations in metropolitan areas that were studied. These are presented in Table 3.

**Table 3:** Statistical results (arithmetic means (AMs) and geometric means (GMs) in Bq/m^3^) of radon and thoron measurements in 34 metropolitan areas across Canada

Metropolitan area	Population*	Results		^222^Rn			^220^Rn		*R*_Tn_
		(*C*_Tn_ > *DL*)	AM	GM	GSD	AM	GM	GSD	*E*_Tn_/(*E*_Rn_ + *E*_Tn_) (%)
Abbotsford-Mission	180,518	52	52	44	2.01	17	13	1.93	5.5
Barrie	197,059	44	63	54	1.81	16	13	1.88	4.5
Brantford	134,203	46	86	58	2.67	21	14	2.23	4.2
Calgary	1,392,609	35	104	90	1.81	11	10	1.70	1.9
Edmonton	1,321,426	46	102	83	1.95	14	12	1.84	2.5
Fredericton	58,220	17	79	51	2.32	203	86	3.19	31.8
Greater Sudbury	164,689	40	110	86	2.02	16	12	1.93	2.5
Guelph	151,984	43	79	63	1.87	14	11	1.86	3.0
Halifax–Dartmouth	495,691	79	123	60	3.09	29	19	2.37	4.1
Hamilton	747,545	45	71	59	1.82	16	12	1.93	3.9
Kelowna	194,882	45	125	92	2.36	16	13	1.96	2.3
Kingston	161,175	44	167	112	2.52	21	15	2.15	2.2
Kitchener–Cambridge–Waterloo	523,894	47	58	52	1.62	15	11	1.93	4.4
London	494,069	38	73	64	1.71	13	11	1.77	3.0
Moncton	144,810	48	71	50	2.29	14	11	1.88	3.4
Montréal	4,098,927	51	117	64	3.01	15	13	1.71	2.2
Oshawa	379,848	54	55	41	2.21	18	13	2.05	5.5
Ottawa—Gatineau	1,323,783	122	102	67	2.35	52	24	3.03	8.5
Peterborough	121,721	48	80	67	1.77	12	11	1.70	2.7
Québec	800,296	41	78	48	2.65	17	11	2.19	3.9
Regina	236,481	27	234	175	2.09	17	12	2.20	1.3
Saguenay	160,980	56	68	42	2.80	17	12	2.04	4.3
Saint John	126,202	62	103	57	2.89	19	13	2.25	3.3
Saskatoon	295,095	39	135	120	1.65	20	16	1.95	2.6
Sherbrooke	212,105	44	145	89	2.42	16	13	1.77	1.9
St. Catharines—Niagara	406,074	55	54	44	2.06	16	14	1.85	5.2
St. John's	205,955	45	70	55	2.10	12	11	1.74	3.1
Thunder Bay	121,621	41	139	83	3.36	19	14	2.05	2.4
Toronto	5,928,040	58	55	44	1.92	12	11	1.70	3.9
Trois-Rivières	156,042	65	42	36	1.84	14	11	1.84	5.8
Vancouver	2,463,431	58	25	21	1.86	12	10	1.67	7.6
Victoria	367,770	56	34	27	1.96	12	10	1.69	6.1
Windsor	329,144	39	115	95	1.89	22	15	2.17	3.4
Winnipeg	778,489	108	177	131	2.22	24	16	2.30	2.4
Canada	24,874,778	1738	84	60	2.20	17	13	1.89	4.0

*DL* detection limit, *E*_*Tn*_ effective dose from thoron (^220^Rn) exposure, *E*_*Rn*_ effective dose from radon (^222^Rn) exposure

*Populations are taken from Census 2016 population (Statistics Canada 2020)

For radon (^222^Rn), the population-weighted AM of 84 Bq/m^3^ is similar to the mean value of 96 Bq/m^3^, which was calculated using all 3215 measurements from 33 metropolitan areas (as reported in Chen et al. 2015), and it is even closer to the population-weighted AM of 82 Bq/m^3^ with long-term radon measurements in a total of 21,818 homes in a recent pooled analysis (Chen 2021). In the present study, the population-weighted GM is found to be 60 Bq/m^3^ with the GSD of 2.20, favourably comparable with the recent analysis from measurements in 21,818 homes, where GM and GSD were calculated as 55 Bq/m^3^ and 2.45 (Chen 2021). This indicates that even relatively limited data from across Canada (such as 1738 measurements in 34 metropolitan areas) can adequately represent radon distribution characteristics in Canada.

For thoron (^220^Rn), the population-weighted AM, GM and GSD are 17 Bq/m^3^, 13 Bq/m^3^ and 1.89, respectively, using only measured *C*_Tn_ above DL. The AM value of 17 Bq/m^3^ is significantly higher than the mean value of 9 Bq/m^3^ which was assessed previously using 3215 measurements and assigning a value of 2 Bq/m^3^ to the 1543 *C*_Tn_ that were less than DL (Chen et al. 2015). Clearly, thoron exposure was significantly underestimated in the previous assessment.

Based on Eqs. (3) and (4), using the measured AM radon and thoron concentrations, annual effective doses were calculated for each metropolitan area and the thoron contribution to the total indoor radon exposure was determined according to Eq. (5). Results are given in the last column of Table 3. Thoron contribution to the radon dose varied widely, ranging from 1.3% in Regina to 32% in Fredericton. The wide variation is mainly due to underlying geology. In an early study by comparison with the results of airborne gamma ray spectrometry survey (Chen et al. 2010), it was demonstrated that the underlying geology was well represented by the average indoor radon and thoron levels. The average indoor radon concentration is higher in areas of higher equivalent uranium concentration in soil, while the average indoor thoron concentration is higher in areas of higher equivalent thorium concentration in the ground. On average (population-weighted), thoron contributes 4.0% of the radiation effective dose due to indoor radon and thoron exposure in Canada, up 1.3% from previous assessment which suggested 2.7% (Chen et al. 2015).

The estimated average indoor thoron concentration in Canada is 17 Bq/m^3^ (population weighted), comparable with the worldwide average indoor thoron level of 15 Bq/m^3^ (0.3 Bq/m^3^ Equilibrium Equivalent Concentration) (UNSCEAR 2000). The estimated average indoor radon concentration of 84 Bq/m^3^ (population weighted) is, however, more than double the worldwide average indoor radon concentration of 40 Bq/m^3^ (UNSCEAR 2000, 2010). It is clear that ongoing efforts are warranted to further reduce the exposure to radon (^220^Rn and ^222^Rn) and effectively reduce the number of radon-induced lung cancer cases.

## Conclusions

Simultaneous radon and thoron measurements were conducted in a total of 3534 residential homes in 34 metropolitan areas covering 71% of Canadian population. While radon concentrations in almost all homes were above the detector’s detection limit, thoron concentrations were measurable in only 1738 homes, possibly due to its short half-life of 56 s. The study confirmed that thoron is log-normally distributed in Canadian homes. In this pooled analysis, which excludes results below detection limit, indoor thoron concentration is characterized by a population-weighted geometric mean of 13 Bq/m^3^ and a geometric standard deviation of 1.89.

Thoron contribution to indoor radon dose varied widely, ranging from 1.3 to 32%. The study indicated that on average, thoron contributes 4% of the radiation dose due to indoor radon and thoron exposure in Canada.

The above analysis only represents statistical averages. The variation characteristics of indoor thoron are similar to indoor radon. Like in the case of indoor radon, indoor thoron concentration can also vary widely, and depends not only on local geology but also on individual house construction and occupants’ living style. Even though, on average, thoron contributes 4% of the radiation dose due to indoor radon and thoron exposure in Canada, indoor thoron level can still be high in some cases. For example, the thoron levels in 45 homes in Fredericton varied from non-detectable to about 2000 Bq/m^3^. The annual effective dose for exposure to thoron concentration of 2000 Bq/m^3^ is about 12 mSv, more than double the annual effective dose for exposure to radon at Canadian guideline level of 200 Bq/m^3^.

Radon (^222^Rn and ^220^Rn) has been identified as the second leading cause of lung cancer after tobacco smoking, and it is the leading cause of lung cancer among non-smokers (WHO 2009; UNSCEAR 2019). It was estimated that more than 3,000 people in Canada die from radon-induced lung cancer each year (Chen et al. 2012b). With increased public awareness of radon-induced lung cancer, people often look at radon potential map and ask questions about the correlation between housing characteristics and indoor radon concentration. However, the fact is that radon levels can vary from one house to another, even if they are similar designs and next door to each other. Therefore, no matter the age, type of construction or where a home is located, the only way to be sure of the radon level in a home is to test. Ongoing efforts are warranted to further reduce the exposure to indoor radon and effectively reduce the number of radon-induced lung cancer cases.
